# Symptomatology, (Co)occurrence and Differential Diagnostic PCR Identification of ‘*Ca.* Phytoplasma solani’ and ‘*Ca.* Phytoplasma convolvuli’ in Field Bindweed

**DOI:** 10.3390/pathogens10020160

**Published:** 2021-02-03

**Authors:** Jelena Jović, Slavica Marinković, Miljana Jakovljević, Oliver Krstić, Tatjana Cvrković, Milana Mitrović, Ivo Toševski

**Affiliations:** 1 Department of Plant Pests, Institute for Plant Protection and Environment, 11080 Zemun, Serbia; slavicamar@gmail.com (S.M.); miljka06@gmail.com (M.J.); oliverk13@yahoo.com (O.K.); tanjacvrkovic@yahoo.com (T.C.); milana.mitrovic@izbis.bg.ac.rs (M.M.); tosevski_ivo@yahoo.com (I.T.); 2 CABI, 2800 Delémont, Switzerland

**Keywords:** bois noir, *Convolvulus arvensis*, duplex SYBR Green real-time PCR, end-point nested PCR, melting point temperature, phytoplasma, reservoir plant, symptoms

## Abstract

Field bindweed (*Convolvulus arvensis*) is one of the major natural plant hosts and reservoirs of ‘*Candidatus* Phytoplasma solani’ (‘*Ca.* P. solani’), the causal agent of plant diseases in diverse agricultural crops, including Bois noir (BN) disease of grapevine. Phylogenetically, the most closely related phytoplasma to ‘*Ca.* P. solani’, the ‘*Ca.* P. convolvuli’, induces disease in field bindweed that is known by its symptoms as bindweed yellows (BY). The occurrence, coinfection and symptoms association of the two phytoplasmas in shared host plants were the subject of this study. Specific primers for the amplification of the elongation factor Tu gene (*tuf*) were developed for the identification of ‘*Ca.* P. convolvuli’ (by conventional nested PCR), as well as primers for simultaneous detection of ‘*Ca.* P. solani’ and ‘*Ca.* P. convolvuli’ by duplex SYBR Green real-time PCR. Among symptomatic bindweed plants, 25 and 41% were infected with a single phytoplasma species, ‘*Ca.* P. solani’ and ‘*Ca.* P. convolvuli’, respectively, while 34% were infected with both phytoplasmas. None of the non-symptomatic control plants carried phytoplasma, while non-symptomatic plants from our previous epidemiological studies in BN-affected vineyards were confirmed to be infected solely with ‘*Ca.* P. solani’. *Stamp* gene typing revealed Rqg50 and Rqg31 ‘*Ca.* P. solani’ genotypes in plants coinfected with ‘*Ca.* P. convolvuli’, while three diverse genotypes (Rqg50, GGY and Rpm35) were identified in a single locality with symptomatic bindweeds infected solely with ‘*Ca.* P. solani’. Variations in symptoms and their association with each of the phytoplasmas are described and documented. The symptom of bushy appearance could be single out as specific for ‘*Ca.* P. convolvuli’ infection, while occurrence of ‘*Ca.* P. solani’ could not be unequivocally associated with specific alterations in infected bindweeds. The results are discussed in the context of the epidemiological and ecological complexity of ‘*Ca.* P. solani’-induced diseases and the relationship between the two phytoplasma relatives in shared host plant.

## 1. Introduction

Field bindweed (*Convolvulus arvensis*) is a climbing herbaceous perennial plant native to Eurasia but widely naturalized in North America and, thus, now considered a cosmopolitan weed. In Serbia, it occurs mainly in open habitats, along road sides, railroads, agricultural fields, and ruderal and degraded natural areas [1]. Field bindweed is one of the major and most widely distributed natural hosts and plant reservoirs of ‘*Candidatus* Phytoplasma solani’ (‘*Ca.* P. solani’) and its planthopper vector *Hyalesthes obsoletus* (Hemiptera: Cixiidae) in agroecosystems and in natural habitats of the Euro-Mediterranean area [2,3,4,5,6,7]. ‘*Ca.* P. solani’ is a member of the 16SrXII-A phytoplasma subgroup, previously known under its trivial name—stolbur phytoplasma [8]. It is the causal agent of several important plant diseases of diverse agricultural crops, among which Bois noir (BN) disease of grapevine is the most widespread and economically most significant [8,9]. Although field bindweeds are often found to be source plants of ‘*Ca.* P. solani’ infection in agroecosystems, the data on symptoms occurrence (presence/absence), variations and characteristics in infected plants are inconsistent and often inconclusive. According to numerous studies, specific symptoms described as “stunting, dwarfism, proliferation, leaf yellowing and/or color alteration” are displayed in the naturally ‘*Ca.* P. solani’-infected *C. arvensis* plants acting as reservoirs and inoculum source for the vector [3,10,11,12,13,14,15,16,17,18]. Conversely, several more recent studies indicate that non-symptomatic plants of *C. arvensis* are a natural source of ‘*Ca.* P. solani’ infection in agroecosystems [6,19,20,21,22,23,24,25]. Furthermore, the phytoplasma that is most closely related to ‘*Ca.* P. solani’ is ‘*Candidatus* Phytoplasma convolvuli’ (‘*Ca.* P. convolvuli’; 16SrXII-H phytoplasma subgroup), which induces specific symptoms in field bindweed, known as bindweed yellows (BY) [26,27,28].

The occurrence of ‘*Ca.* P. convolvuli’-induced disease of field bindweed was first recorded in Italy between 1994 and 1996 [26] and was associated with symptoms described as stunting and yellowing of *C. arvensis* plants that were similar to those documented in symptomatic ‘*Ca.* P. solani’-infected bindweeds from BN-affected vineyards in Germany [10]. However, Marcone and coauthors [26] clearly demonstrated that the identified phytoplasma infecting bindweeds in Italy was different from but closely related to stolbur (‘*Ca.* P. solani’). Later, in a review on the classification of phytoplasma groups based on the 16S rRNA gene, bindweed phytoplasma isolates were recognized as a new, Italian bindweed stolbur (IBS) phytoplasma group [27]. In 2012, the same phytoplasma was defined as the causal agent of bindweed yellows (BY) symptoms in affected *C. arvensis* plants and was characterized by 16S rRNA gene sequencing and described as a novel phytoplasma taxon, ‘*Ca.* P. convolvuli’ [28]. To date, its occurrence has been reported in Germany, Austria, Bosnia and Herzegovina, Serbia, Georgia and Poland [19,28,29,30]. The only known host of ‘*Ca.* P. convolvuli’ is *C. arvensis*, apart from a single record of its presence in a symptomless *Urtica dioica* found in BN-affected vineyards in Austria [19].

Given the epidemiological and ecological complexity of ‘*Ca.* P. solani’-induced diseases [6,7,8,19,20,21,22,23,31,32,33,34,35,36,37,38,39,40,41,42] and its evolutionary relatedness to ‘*Ca.* P. convolvuli’, the focus of this study was to analyze the (co)occurrence of the two pathogens in shared host plant and related symptoms. The main aims were to reliably identify and characterize phytoplasmas in symptomatic and/or non-symptomatic field bindweeds and to document symptom variations associated with each of the two phytoplasma taxa in the field. The study was focused on natural or ruderal habitats, distant from vineyards or other ‘*Ca.* P solani’-affected agricultural fields to minimize effects caused by herbicide treatments and avoid infection pressure deriving from other agroecosystems. To test if hidden (non-symptomatic) infections with ‘*Ca.* P. convolvuli’ or co-infections with ‘*Ca.* P. solani’, the analyses included non-symptomatic populations of *C. arvensis* collected in BN-affected vineyards during our previous epidemiological studies. A protocol for fast, precise and cost-effective identification and differentiation of both phytoplasmas in *C. arvensis* was designed and tested to provide tools for future screenings and epidemiological surveys.

## 2. Results

### 2.1. Occurrence of ‘Ca. P. solani’ and ‘Ca. P. convolvuli’ in Symptomatic Field Bindweeds

A three-year study (2018–2020) on the occurrence of symptomatic *C. arvensis* in natural and ruderal habitats of Serbia (Table 1) revealed that ‘*Ca.* P. solani’ and ‘*Ca.* P. convolvuli’ were present in affected plants (Table 2). A total of 68 symptomatic and 66 non-symptomatic control plants were collected at four localities in eastern and central Serbia. All symptomatic plants were found to be infected with either ‘*Ca.* P. solani’ or ‘*Ca.* P. convolvuli’ or with both phytoplasmas in mixed infection, while all control plants were phytoplasma-free. Initial ‘*Ca.* P. convolvuli’ identification was performed by 16S rRNA and *tuf* gene sequencing, while later diagnostic protocols included ‘*Ca.* P. solani’-specific *stamp* nested PCR protocol [43], a ‘*Ca.* P. convolvuli’-specific *tuf* nested PCR protocol designed in this study and novel duplex SYBR Green-based real-time PCR on the *tuf* gene for simultaneous detection of ‘*Ca.* P. solani’ and/or ‘*Ca.* P. convolvuli’.

A population of symptomatic bindweed plants infected solely with a single phytoplasma species was detected only at one location, Kladovo, in the eastern-most part of Serbia, harboring ‘*Ca.* P. solani’ (Table 2). In the other locations, two in eastern Serbia, Donji Milanovac and Štubik, and the locality Deč in central Serbia, symptomatic plants were infected with both phytoplasmas, frequently in mixed infection (overall 41% of symptomatic plants sampled at these locations). In total, among all symptomatic bindweeds in all four localities, 25% (17/68) were infected with ‘*Ca.* P. solani’ and 41% (28/68) with ‘*Ca.* P. convolvuli’, while 34% (23/68) were infected with both phytoplasmas (Table 2).

**Figure 1:**
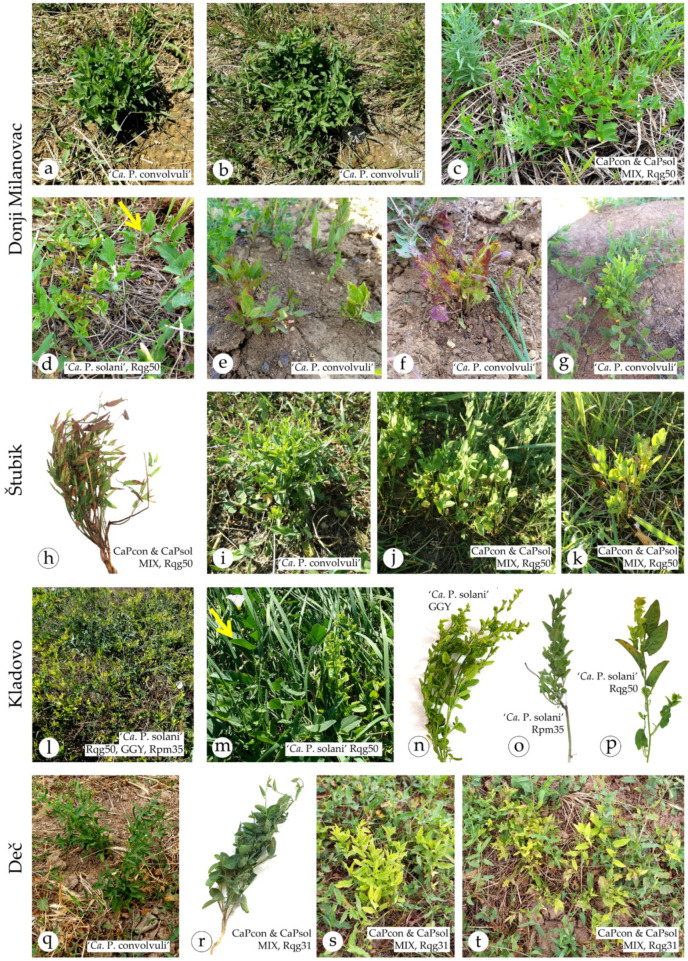
Variations in symptoms in phytoplasma-infected field bindweed (*Convolvulus arvensis*) observed in this study. The locality of plant origin is indicated on the left side of the figure: (**a**–**g**) Donji Milanovac, (**h**–**k**) Štubik, (**l**–**p**) Kladovo and (**q**–**t**) Deč. On each image is denoted the phytoplasma(s) detected in the symptomatic plant (‘*Ca.* P. solani’ and/or ‘*Ca.* P. convolvuli’) and the *stamp* genotype in case of (co)infection with ‘*Ca.* P. solani’. Non-symptomatic field bindweed next to the symptomatic plants are indicated by yellow arrows on images (**d**,**m**).

The first observation of symptomatic field bindweeds was made in August 2018 during a survey on phytoplasma vectors in natural habitats of Serbia [42], at location Donji Milanovac, on abandoned pasture slopes situated between the Great and Small Kazan of the Iron Gates gorge on the Danube River. These plants had characteristic symptoms of bushy appearance, elongated leaves and discrete yellowing (Table 2, Figure 1a,b) and were infected with ‘*Ca.* P. convolvuli’ based on 16S rRNA gene sequencing and BLASTn comparison (100% identity with referent strain BY-S57/11, GenBank acc. no. JN833705). *Tuf* gene sequencing confirmed this finding, since the obtained sequences were nearly identical (1 nt difference) to that of a ‘*Ca.* P. convolvuli’ isolate from Austria [19] (KJ469710). This location was sampled for the following two years, which led to the observation of diverse symptoms, as well as the occurrence of ‘*Ca.* P. solani’ in a primary or double infection with ‘*Ca.* P. convolvuli’. In 2018, all plants were infected with ‘*Ca.* P. convolvuli’ (Figure 1a,b); in 2019, four out of 16 were ‘*Ca.* P. solani’-infected (Figure 1d), six were ‘*Ca.* P. convolvuli’-infected, and six exhibited double infection. In 2020, all analyzed plants were primary infected with ‘*Ca.* P. convolvuli’ (Table 2). All symptomatic plants that were infected or coinfected with ‘*Ca.* P. convolvuli’ at this location had symptoms of bushy appearance (Figure 1a–c,e–g), while plants infected solely with ‘*Ca.* P. solani’ had symptoms of undersized leaves and marginal reddening of leaves (Figure 1d). Symptomatic ‘*Ca.* P. convolvuli’-infected bindweed plants sampled in June 2020 had symptoms of yellowing, leaf veins reddening and shoot proliferation (Figure 1e–g), in addition to bushy appearance.

A similar situation was found at location of Štubik (eastern Serbia), on the mowed meadow next to the regional road. All symptomatic plants were ‘*Ca.* P. convolvuli’-infected and all had symptoms of bushy appearance, sometimes accompanied by symptoms of leaf veins reddening, yellowing and shoot proliferation (Table 2, Figure 1h–k). Many of the symptomatic plants were mixed infected with ‘*Ca.* P. solani’; however, no clear difference in symptoms was observed between plants infected solely with ‘*Ca.* P. convolvuli’ and those coinfected with ‘*Ca.* P. solani’.

At locality Kladovo, a ruderal site situated on the Danube River bank in front of the medieval castle of Fetislam, homogenously covered with field bindweeds, we observed distinctive yellow patches of symptomatic plants (Figure 1l). These symptoms differed from previously recorded in pronouncedly undersized leaves and yellowing, internode shortening, and especially secondary shoot proliferation (Figure 1m–p). All symptomatic plants were found to be primary infected with ‘*Ca.* P. solani’.

Locality Deč in central Serbia hosted symptomatic *C. arvensis* plants infected with both phytoplasmas in the form of primary or double infection. Symptoms included a bushy appearance (Figure 1q,r), sometimes with pronounced yellowing (Figure 1s,t).

### 2.2. Application of Novel ‘Ca. P. convolvuli’-Specific Nested PCR Diagnostic Protocol

**Figure 2:**
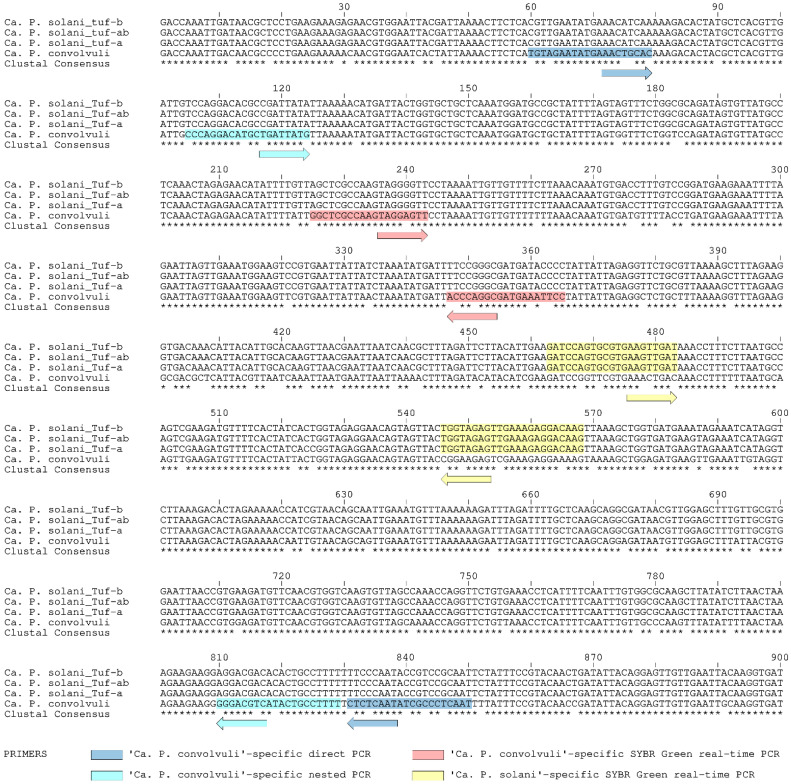
Position and sequence specificity of novel primers on the elongation factor Tu gene (*tuf*) multiple sequence alignment of three major representative *tuf* types of ‘*Ca.* P. solani’ and of ‘*Ca.* P. convolvuli’. The sequence covers 900-bp region amplified using the primer pair TufAYf/TufAYr [45]. The novel primers are designed for conventional (end-point) nested PCR and for SYBR Green-based real-time PCR for the detection and differential identification of ‘*Ca.* P. solani’ and of ‘*Ca.* P. convolvuli’ in field bindweed.

**Figure 3:**
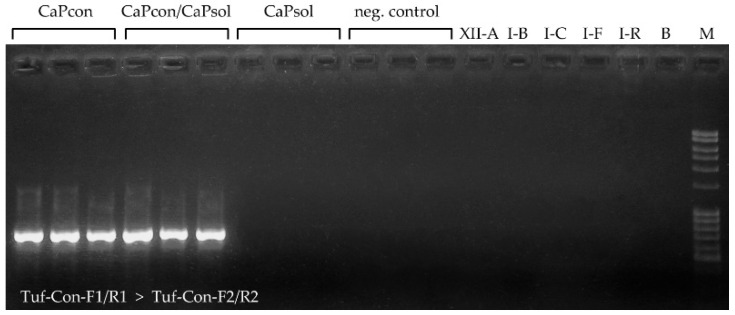
Agarose gel electrophoresis of ‘*Ca.* P. convolvuli’-specific nested PCR amplicons obtained using the Tuf-Con-F1/R1 primer pair (791-bp long fragment, Ta = 55 °C) followed by Tuf-Con-F2/R2 primers (725 bp, Ta = 65 °C). The samples are designated as follows: ‘*Ca.* P. convolvuli’-infected bindweed samples (CaPcon), ‘*Ca.* P. convolvuli’ and ‘*Ca.* P. solani’ coinfected bindweed samples (CaPcon/CaPsol), ‘*Ca.* P. solani’-infected bindweed samples (CaPsol); non-symptomatic bindweed isolates negative for any phytoplasmas as determined by 16S rRNA analysis (neg. control); XII-A, I-B, I-C, I-F, I-R: ‘*Ca.* P. solani’ and ‘*Ca.* P. asteris’ isolates from previous studies [6,23,39,40,42,44]; B: negative control containing PCR mixture and molecular grade water; M: DNA ladder 100 bp (Serva).

A conventional nested PCR protocol was designed for the *tuf* gene encoding elongation factor Tu and tested as a diagnostic method for selective amplification and identification of ‘*Ca.* P. convolvuli’ occurrence in bindweed (Figure 2 and Figure 3). The presence of ‘*Ca.* P. convolvuli’ was determined in 51 out of 68 symptomatic plants by yielding PCR product of the expected size (725-bp; Table 3, Figure 3). Sequence analyses, followed by BLASTn comparison of ten randomly selected nested PCR products, confirmed their identity. No diverse ‘*Ca.* P. solani’ and ‘*Ca.* P. asteris’ isolates [6,23,39,40,42,44], tested as controls in specificity assays, gave any product, nor did non-symptomatic bindweed isolates or ‘*Ca.* P. solani’-infected symptomatic bindweeds collected in this study (Figure 3).

Analyses of DNA material from non-symptomatic ‘*Ca.* P. solani’-infected populations of *C. arvensis* collected in BN-affected vineyards in North Macedonia, Montenegro and Serbia [6,20,23] using the novel ‘*Ca.* P. convolvuli’-specific nested PCR assay revealed that all of the samples were negative for ‘*Ca.* P. convolvuli’ infection.

### 2.3. Application of Novel Duplex SYBR Green-Based Real-Time PCR for Simultaneous Detection and Differentiation of ‘Ca. P. solani’ and ‘Ca. P. convolvuli’

**Figure 4:**
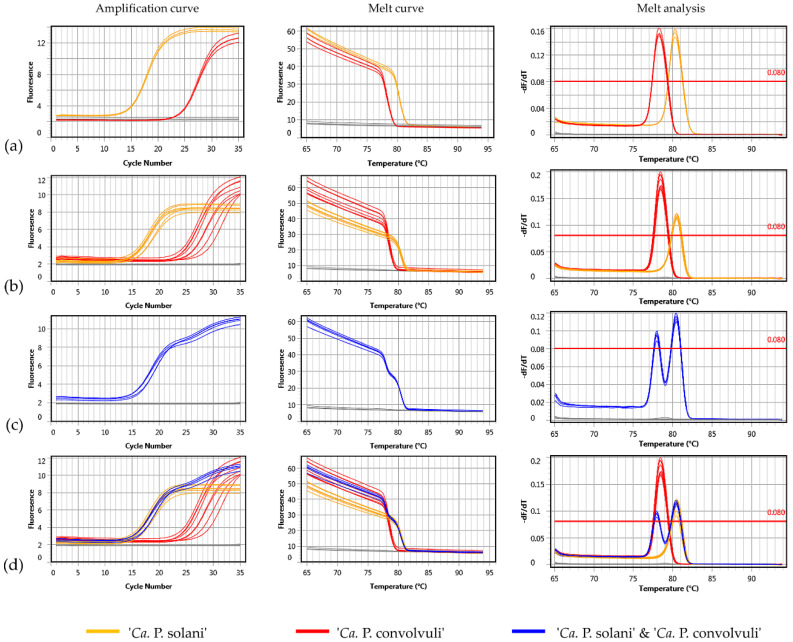
Single (**a**) and duplex (**b**–**d**) SYBR Green-based real-time PCR amplification characteristics using novel ‘*Ca.* P. solani’- and of ‘*Ca.* P. convolvuli’-specific primer pairs Tuf-Sol-Fq/Rq and Tuf-Con-Fq/Rq: amplification curve, melt curve and melt analysis for single-infected and mixed-infected samples. In the images, (**a**) single infected samples are presented when amplified using a primer mixture with an equal concentration of primer pairs specific for ‘*Ca.* P. solani’ or ‘*Ca.* P. convolvuli’. Images (**b**–**d**) show the melting and amplification characteristics in primer mixture containing 4-fold more ‘*Ca.* P. convolvuli’-specific primers than ‘*Ca.* P. solani’-specific ones. Among them, images (**b**) show samples infected with a single phytoplasma species, images (**c**) show samples coinfected with ‘*Ca.* P. solani’ and ‘*Ca.* P. convolvuli’, and images (**d**) show both types of samples.

A diagnostic protocol for the simultaneous identification of both phytoplasmas in bindweeds was designed for selective coamplification of ‘*Ca.* P. solani’ and ‘*Ca.* P. convolvuli’ using a SYBR Green-based real-time PCR approach. The primer pair Tuf-Con-Fq/Rq was designed to match and amplify a 141-bp long *tuf* gene fragment of ‘*Ca.* P. convolvuli’ with 31% GC content resulting in a uMelt Quartz predicted melting point temperature (Tm) of 78 °C, while primers Tuf-Sol-Fq/Rq were designed to amplify a 106-bp long *tuf* gene fragment of ‘*Ca.* P. solani’ containing 41% GC nucleotides resulting in a predicted Tm of 81 °C (Figure 2, Table 3). The protocol was first tested in separate single-primer set (singleplex) reactions and later applied as duplex PCR using a mix of ‘*Ca.* P. solani’-specific and ‘*Ca.* P. convolvuli’-specific primers (Figure 4). All 134 *C. arvensis* samples collected in this study (68 symptomatic and 66 control plants) and 122 samples from our previous studies in BN-affected vineyards [6,20,23] underwent duplex SYBR Green-based real-time PCR analyses.

The performance of the Tuf-Con-Fq/Rq and Tuf-Sol-Fq/Rq primer sets was tested in singleplex reactions using LinRegPCR [46] and the standard curve analysis method against 10-fold serial dilutions of bindweed samples in which ‘*Ca.* P. convolvuli’ or ‘*Ca.* P. solani’ was previously identified by conventional nested PCR protocols, which provided data on efficiency values (E) and quality measures of the linear regression applied to calculate amplification efficiency (R^2^). ‘*Ca.* P. convolvuli’-specific primers efficiency values were 0.93 ± 0.02, R^2^ > 0.999, and those for ‘*Ca.* P. solani’-specific primers were 0.91 ± 0.08, R^2^ > 0.999. The reproducibility between sample replicates and runs was also high, with low standard deviation (SD) for the value of the mean Ct (threshold cycle) (Table 4). The Ct values for all diverse samples infected with ‘*Ca.* P. solani’ were 14.8 ± 2.9 (12.7–16.9) in routine diagnostics using 50 ng of DNA extract per reaction, while for ‘*Ca.* P. convolvuli’ samples, the Ct values under the same conditions were 20.4 ± 9.9 (13.3–27.4). For the majority of ‘*Ca.* P. convolvuli’ samples, the Ct value was between 20 and 23, and due to the 4-fold higher concentration of ‘*Ca.* P. convolvuli’-specific primers was used in duplex reaction. Only bindweed samples collected in June 2020, infected solely with ‘*Ca.* P. convolvuli’ had higher Ct values, between 13.29 and 17.62 (15.46 ± 3.06). According to the standard curve analysis method the performance of the Tuf-Con-Fq/Rq primers amplification was 94%, R^2^ > 0.999 (slope = −3.46x + 29.13) and that of the Tuf-Sol-Fq/Rq primers was 92%, R^2^ > 0.998 (slope = −3.514x + 29.46).

All symptomatic bindweeds infected with ‘*Ca.* P. solani’ and/or ‘*Ca.* P. convolvuli’ from our study were confirmed to be infected by associated phytoplasma(s) using the SYBR Green-based real-time PCR approach. The method proved to be very fast, reliable and elegant, since double infections can often be determined during the amplification process on the amplification curve (i.e., two plateaus, Figure 4c), while are definitely confirmed by melting curve analyses (Tm of ‘*Ca.* P. convolvuli’ 78.26 °C ± 0.11 and that of ‘*Ca.* P. solani’ 80.38 °C ± 0.12; Table 3, Figure 4).

Analyses of non-symptomatic ‘*Ca.* P. solani’-infected populations of *C. arvensis* originating from BN-affected vineyards collected during our previous studies [6,20,23] using the novel duplex SYBR Green-based real-time PCR diagnostic protocol confirmed that they were free of ‘*Ca.* P. convolvuli’. Additionally, identification of ‘*Ca.* P. solani’ in these samples occurred in 14% (7 out of 50), 25% (15 out of 60) and 42% (5 out of 12) of bindweeds collected in the surroundings of BN vineyards in North Macedonia, Montenegro and Serbia, respectively. These results confirmed previous diagnostics of ‘*Ca.* P. solani’ infection using conventional nested PCR targeting the *tuf*, *vmp1*, and *stamp* genes [6,20,23].

**Figure 5:**
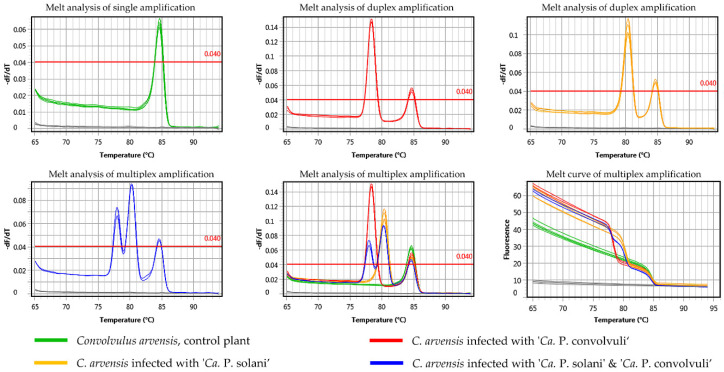
Melt curve analysis in multiplex SYBR Green-based real-time PCR using novel ‘*Ca.* P. solani’- and of ‘*Ca.* P. convolvuli’-specific primer pairs Tuf-Sol-Fq/Rq and Tuf-Con-Fq/Rq and the primers for the 28S rDNA gene [48] amplifying *Convolvulus arvensis* as endogenous control.

Assays with primers targeting 28S rRNA of *C. arvensis* as an endogenous control (EC) yielded amplification in every reaction for each bindweed sample (Ct = 12.98 ± 0.55, E = 0.95 ± 0.001, R^2^ > 0.999). The melting point temperature predicted by uMelt Quartz for the targeted segment of the 28S rRNA plant gene was 84.5 °C, while the Tm mean ± SD obtained experimentally for all samples was 84.35 °C ± 0.1. The Tm of EC indicated that it could be discriminated in the same reaction together with both phytoplasmas tentatively infecting bindweeds. Experimental testing of the multiplex reaction with three primer pairs (‘*Ca.* P. solani’-specific, ‘*Ca.* P. convolvuli’-specific and 28S as an internal control) revealed amplification of three fragments with distinctive melting curve temperatures in samples coinfected with ‘*Ca.* P. solani’ and ‘*Ca.* P. convolvuli’ (Figure 5). Since Tm can vary due to the chemistry of SYBR Green PCR mix, as well as between different qPCR instruments, the capability of Tm differentiation was tested in multiplex amplification using SsoAdvanced™ Universal SYBR^®^ Green Supermix (Bio-Rad Laboratories, Inc., Hercules, CA, USA) and KAPA SYBR FAST qPCR Kit Master Mix (Kapa Biosystems), both analyzed by micPCR^©^ software Version 2.6.4 (Bio Molecular Systems, Upper Coomera, QLD, Australia). The Tm values obtained using the Bio-Rad Kit were approximately 78 °C for Tuf-Con-Fq/Rq amplicons of ‘*Ca.* P. convolvuli’, 80 °C for Tuf-Sol-Fq/Rq of ‘*Ca.* P. solani’ and 84 °C for EC (Figure 5). For the KAPA Kit, the Tm values of the same targets were 76.5 °C for ‘*Ca.* P. convolvuli’, 78.5 °C for ‘*Ca.* P. solani’ and 82 °C for EC (data not shown). Hence, differences among the Tm values of the three diverse targets were constant and comparable and, thus, the protocol could be applied as a triplex in a single reaction tube.

### 2.4. ‘Ca. P. convolvuli’ and ‘Ca. P. solani’ Genotype Strains

The 28 symptomatic bindweed samples primary infected with ‘*Ca.* P. convolvuli’ were genotyped by sequence analysis of the 16S rRNA and *tuf* genes. Nucleotide sequence data comparison found no variability between isolates on both genes from all three locations. The sequence comparison revealed complete identity of the 16S rRNA gene with the reference ‘*Ca.* P. convolvuli’ strain BY-S57/11 from Serbia [28] and a single nucleotide polymorphism in the *tuf* gene sequence compared to the available sequence data of isolate from Austria [19]. Other more variable genes of ‘*Ca.* P. convolvuli’ which would be of significance for epidemiological characterization, i.e., the *secY* gene encoding a translocase protein [12], variable membrane protein *vmp1* [49] and antigenic membrane protein gene *amp* [43] were attempted to be analyzed. Primers and protocols designed for ‘*Ca.* P. asteris’ or ‘*Ca.* P. solani’ were applied; however, these yielded no amplification, probably due to sequence uniqueness and therefore the inability of current primers and protocols to amplify diversified strains of closely related ‘*Ca.* Phytoplasma’ species.

The 17 symptomatic *C. arvensis* samples primary infected with ‘*Ca.* P. solani’ and 23 samples coinfected with both phytoplasmas were characterized by the *stamp* gene of ‘*Ca.* P. solani’. Genotyping revealed the Rqg50 and Rqg31 *stamp* variants in plants on three locations where bindweeds were coinfected with ‘*Ca.* P. convolvuli’ (Table 2). All ten ‘*Ca.* P. solani’-infected bindweeds (primary and coinfected) at location Donji Milanovac, as well as eight ‘*Ca.* P. solani’/‘*Ca.* P. convolvuli’ coinfected plants at location Štubik, harbored a single genotype, namely, Rqg50. Correspondingly, the ten bindweeds at location Deč, primary and coinfected, all harbored the same genotype, Rqg31. However, in a single locality with symptomatic bindweeds infected solely with ‘*Ca.* P. solani’ (Kladovo, eastern Serbia), three diverse genotypes (Rqg50, GGY and Rpm35) were identified (Table 2, Figure 1).

## 3. Discussion

Epidemiology and routes of transmission of ‘*Ca.* P. solani’ and associated diseases of agricultural crops are complex and dependent upon weed host-plant species that serve as a shared host for the vector and the pathogen. Furthermore, the main epidemiological transmission pathways are driven by host-plant specialized populations of the planthopper vector *H. obsoletus* e.g., [4,6,7,31]. To date, four routes of transmission (epidemiological cycles) of ‘*Ca.* P. solani’ are evidenced to be vectored by different plant-specialized populations of *H. obsoletus*, mainly related to the BN disease of grapevine [2,3,6,23,31,50]. Each of the transmission routes is associated with a specific weedy host plant shared by the vector and the pathogen: *Urtica dioica* (stinging nettle), *C. arvensis* (field bindweed), *Vitex agnus-castus* (monk’s pepper) and *Crepis foetida* (stinking hawk’s-beard). However, unique for the field bindweed is that it is the only one among listed reservoir plants known to express disease-like symptoms due to ‘*Ca.* P. solani’ occurrence. The results of our study confirm this observation but raise questions regarding the pathogen(s) involved in symptoms appearance since both ‘*Ca.* P. solani’ and ‘*Ca.* P. convolvuli’ were identified in symptomatic bindweed plants, often as a double infection.

Documented symptoms in field-collected bindweeds from our study appeared variable, while each of the two detected phytoplasmas could not be directly or unequivocally associated with specific alterations in symptomatic plants (Figure 1). None of the tested non-symptomatic plants, either collected as controls in this study or as test plants in previous studies on BN epidemiology [6,20,23], gave amplification with ‘*Ca.* P. convolvuli’-specific primers (Table 3). Thus, ‘*Ca.* P. convolvuli’-infected plants were always symptomatic, in our study and in all previous studies [19,26,28,29]. The bushy appearance as a common symptom underlying ‘*Ca.* P. convolvuli’ infection in bindweeds can be singled out, but this symptom can also be accompanied by yellowing (discrete or more pronounced), leaf vein reddening and/or shoot proliferation (Table 2). Conversely, up to this study, all of our previous surveys of bindweeds as a ‘*Ca.* P. solani’ reservoir plants in BN-affected vineyards resulted only in the identification of individuals that carried infection without any symptoms [6,20,23]. However, the current study was focused on symptomatic *C. arvensis* plants and, hence, did provide evidence of ‘*Ca.* P. solani’ occurrence in symptomatic bindweeds, either in primary infection or coinfection with ‘*Ca.* P. convolvuli’. This is in agreement with numerous studies reporting the symptomatic occurrence of ‘*Ca.* P. solani’ in bindweeds e.g., [3,10,11,12,16], but it is questionable whether the symptoms are primarily associated with ‘*Ca.* P. solani’ or are caused by (co)infection with closely related ‘*Ca.* P. convolvuli’ and dependent upon competition and balance between the two pathogens. One could also argue that natural reservoir plants should be non-symptomatic due to co-evolutionary adaptations among insect-vector, pathogen and the shared host-plant, as would be the case of bindweeds as ‘*Ca.* P. solani’ reservoir plant. These questions and hypotheses should be tested and evaluated in controlled laboratory conditions; however, field surveys on the occurrence of the two pathogens and their frequencies in diseased, symptomatic plants can provide an indication of underlying causes.

To obtain reliable and verifiable data on the presence and association of closely related phytoplasmas in a common host plant, field bindweed, specific and sensitive tools for detection and differentiation are needed. ‘*Ca.* P. convolvuli’ is genetically very similar to ‘*Ca.* P. solani’, especially the 16S rRNA gene with which it shares 97.9% sequence identity (based on the F2n/R2 delineated region of reference strains AF248959 and JN833705) [8,28]. It is also closely related to yet another widely distributed and common phytoplasma species, ‘*Ca.* Phytoplasma asteris’ of the 16SrI-B subgroup (reference strain 16S sequence acc. no. M30790) [51] with which it shares 96.9% 16S rRNA sequence identity. Furthermore, the most commonly used method for phytoplasma identification in field-collected material is conventional nested PCR using phytoplasma universal primers targeting the 16S rRNA gene [52]. This can cause problems in the identification of two (or more) phytoplasmas in mixed infection, and could lead to false association of symptoms and causative agent of the disease. In a situation like this, identification of the pathogen(s) present in a symptomatic plant depends on a specificity of primers and protocols, and competition between different target DNAs in the sample; hence, some of the pathogens could remain hidden in a process of detection. It seems that this could be the case for ‘*Ca.* P. solani’ and ‘*Ca.* P. convolvuli’ in field bindweeds, especially because another commonly used protocol for stolbur phytoplasma identification based on the *tuf* gene [31,45] successfully amplifies three phytoplasmas: ‘*Ca.* P. asteris’, ‘*Ca.* P. solani’ and ‘*Ca.* P. convolvuli’ [19,31,45]. Although it was shown that ‘*Ca.* P. convolvuli’ can be differentiated from ‘*Ca.* P. solani’ using *Hpa*II RFLP analysis based on the *tuf* gene amplicons [19], this protocol contributes only to the differentiation of two phytoplasmas but not to their initial detection. Another issue to consider when observing ‘*Ca.* P. solani’-bindweed pathosystem is that ‘*Ca.* P. convolvuli’ was mostly neglected in field surveys (was not searched for), probably due to its absence in commercially valuable agricultural crops (although its presence was not searched for there either) and/or because of the frequent occurrence of ‘*Ca.* P. solani’ in diverse crops and weedy plants within agroecosystems and surrounding natural areas of Europe. Hence, ‘*Ca.* P. convolvuli’ was only sporadically found in bindweeds during surveys for ‘*Ca.* P. solani’ or ‘*Ca.* P. asteris’ reservoir plants using primers and protocols designed and adapted for stolbur and aster yellows phytoplasma detection [19,29,30]. This indicates that distribution, occurrence and frequency of ‘*Ca.* P. convolvuli’ in natural ecosystems and agroecosystems is probably highly underestimated.

Due to all of the abovementioned issues regarding the reliable identification of ‘*Ca.* P. convolvuli’ and ‘*Ca.* P. solani’ in shared host plants, novel molecular identification protocols were developed and tested for their fast, precise and cost-effective detection and differentiation in field bindweeds. The protocol of conventional end-point nested PCR enables selective identification and targeted search for ‘*Ca.* P. convolvuli’ in the field surveys. The protocol of duplex (and multiplex) SYBR Green real-time PCR enables fast and practical simultaneous detection and discrimination of both phytoplasmas by analyzing melting curves of amplified fragments. Recently, a similar protocol involving real-time PCR melting curve analysis of *tuf* gene amplicons was developed for the differentiation of two main ‘*Ca.* P. solani’ epidemiological strains, tuf-a and tuf-b [53]. However, for strain differentiation, which differs by only a few nucleotides, an HRM (high-resolution melting) approach was needed, which is more demanding in terms of the cost of analysis and equipment requirements. In contrast, by developing protocol for the identification and differentiation of two closely related but divergent phytoplasma species in our study, design of primers was possible in almost any segment of the *tuf* gene. Hence, the two segments with a 10% difference in GC content were selected for primers binding sites, which enabled differentiation of the amplicon melting temperatures using standard real-time PCR machine software for fluorescence acquisition and SYBR Green dye and thus made the protocol cost-effective and easy to adopt and use. The development of this novel identification protocol should enable and promote a wider search for ‘*Ca.* P. solani’ and ‘*Ca.* P. convolvuli’ (co)infection in natural ecosystems and agroecosystems. The protocol and associated species-specific primers could further be tested and adopted for quantitative real-time PCR in terms of ‘*Ca.* P. solani’ and ‘*Ca.* P. convolvuli’ quantification (absolute and relative); however, this would require further standardization of the protocol, including determination of LOD (limit of detection) and LOQ (limit of quantification) following MIQE guidelines [54].

In the context of epidemiological and ecological complexity of ‘*Ca.* P. solani’-induced diseases, symptom occurrence in reservoir plants is relevant for understanding the relationship between the two phytoplasma relatives in shared host plant. For ‘*Ca.* P. convolvuli’, the situation seems clear; this pathogen induces disease-like symptoms in its host plant. However, the situation with ‘*Ca.* P. solani’ infection in field bindweeds is far from elucidated. Plants can be either symptomatic or non-symptomatic while infected at similar rates and frequencies e.g., [3,6,10,12,14,18,21,22,23,24]. In our study, only at locality Kladovo symptomatic bindweed plants were infected solely with ‘*Ca.* P. solani’. The observed symptoms in these plants were distinguishably different from those in the other three locations by pronounced yellowing, prominently undersized leaves, shortening of internodes and proliferation of secondary shoots producing yellow, undersized leaves. Similar symptoms were previously recorded in control transmission experiments of “stolbur virus” using *H. obsoletus* specimens collected on *C. arvensis*, *Amaranthus retroflexus* and *Zea mays* var. *saccharata* in central Serbia in 1966 (photo-documented and described in Aleksić et al., 1967) [55]; unfortunately, no precise details on vectors’ host plant were given for this experiment and, at the time, the identification of the pathogen associated with the disease was commonly performed primarily based on observed symptoms, without proper identification. However, symptoms observed in the Kladovo location even more closely resembled the symptoms obtained in a single bindweed experimental plant when the ‘*Ca.* P. solani’ *tuf*/*stamp*/*vmp1* genotype tuf-b/STOL/V2-TA was transmitted to it by the *H. obsoletus* population originating from *Crepis foetida* (photodocumented and described in Kosovac, 2018) [56]. Since *C. foetida*-specialized populations of the vector are proven to be genetically, ecologically and epidemiologically divergent and different from those associated with *C. arvensis* [7,23], one can hypothesize that ‘*Ca.* P. solani’-induced symptoms in the field bindweed, when occurring in primary infection, are actually a consequence of initial transmission by other ‘*Ca.* P. solani’ vector not adapted to a bindweed-sourced pathosystem. The diversity of ‘*Ca.* P. solani’ *stamp* genotypes identified at the Kladovo location (Rqg50, GGY and Rpm35) also contribute to this assumption by indicating possible multiple vectors due to the known occurrence of these genotypes in vectors of other ‘*Ca.* P. solani’ pathosystems [6,20,23,39,40].

The hypothesis on the interference of alternative vectors with bindweed-‘*Ca.* P. solani’-*H. obsoletus* pathosystem, as well as the previous one on the ‘*Ca.* P. convolvuli’ as a primary causative pathogen of symptom appearance in bindweeds, need testing but one can again argue that effective natural reservoir plants should be co-evolutionarily adapted to their associated insect vector and the pathogen they source. The presented results clearly indicate the coexistence of two phytoplasma species inside a single plant system with a different level of detection using traditional protocols of identification, where one of the pathogens could be masked. At this moment, it is difficult to predict the epidemiological significance of ‘*Ca.* P. convolvuli’ as a pathogen, and that is why additional study is needed to clarify the epidemiological significance of both phytoplasmas solely or in its combined occurrence in the environment.

## 4. Materials and Methods

### 4.1. Plant Sampling

Field surveys for symptomatic *C. arvensis* were performed during a period of three years, from 2018 to 2020. Surveyed localities were chosen to be in natural or ruderal habitats, away from vineyards or other ‘*Ca.* P solani’-affected agricultural fields to rule out herbicide application or reinfection from agroecosystem as the cause of symptoms and pathogen occurrence in field bindweeds. Samplings were made from June to September at a total of four localities in eastern and central Serbia (Table 1). From each locality, at least 12 symptomatic plants were collected, chosen by the variations in symptoms and by a distance between plants of at least 2 m to avoid recollection of single plant shoots. Each sampled symptomatic bindweed plant was photo-documented in the field or in the laboratory prior to preparation for DNA extraction. Furthermore, at least 12 non-symptomatic control plants were sampled per locality, from a minimum of 20 m apart of the patches with symptomatic bindweeds.

A DNA material from non-symptomatic ‘*Ca.* P. solani’-infected populations of *C. arvensis* collected in phytoplasma-affected vineyards during our previous research studies on BN epidemiology conducted in North Macedonia, Montenegro and Serbia [6,20,23] was included in the analyses. This was done to test the possibility of hidden (non-symptomatic) occurrence of ‘*Ca.* P. convolvuli’ or cooccurrence with ‘*Ca.* P. solani’.

### 4.2. DNA Extraction, Initial Phytoplasma Identification and Sequencing

Total DNA was extracted from one gram of leaf midribs and petioles of symptomatic and non-symptomatic bindweed samples using a previously reported CTAB protocol [57]. The final total DNA pellet was resuspended in 100 μL of TE buffer. Extracted DNA was kept at −20 °C until further analysis. The quality and concentrations of the obtained DNA extracts were determined using a NanoPhotometer^®^ N60 spectrophotometer (Implen, GmbH, Munich, Germany). The concentration of DNA in the extracts ranged from 950 ng/μL to 1500 ng/μL; hence, all samples were diluted in molecular grade water to a final concentration of 25 ng/μL nucleic acids.

Initial phytoplasma identification in symptomatic bindweeds, collected in 2018, was performed by nested PCR amplification of the 16S rRNA gene and confirmed by amplification of the *tuf* gene (which encodes elongation factor Tu). The reference isolate of ‘*Ca.* P. solani’ isolated from experimentally infected *Catharanthus roseus* with phytoplasma transmitted by *Hyalesthes obsoletus* from Serbia was used as a positive control in all amplification reactions. 16S rRNA gene amplification was performed with the phytoplasma generic primers P1/P7 in direct PCR [58,59] followed by P1A/P7A [60] and F2n/R2 [61] in nested PCR according to previously described reaction conditions [60]. *Tuf* gene amplification was performed using the Tuf1f/r primer pair for direct and TufAYf/r primers for nested PCR [45] following a formerly described thermal protocol [31]. The obtained amplicons of the expected size for both genes were sequenced with the primers used for nested amplifications. Sequencing was performed by Macrogen Europe (Amsterdam, The Netherlands). Obtained sequences were edited using FinchTV v. 1.4.0 (https://digitalworldbiology.com/FinchTV) and assembled using the Clustal W program integrated into MEGA 7 software [62]. Initial phytoplasma identification was performed by sequence comparison using a BLASTn algorithm (https://blast.ncbi.nlm.nih.gov).

### 4.3. Molecular Typing of ‘Ca. P. convolvuli’ Strains

The 16S rRNA and *tuf* genes were genotyped for all bindweed samples primary infected with ‘*Ca.* P. convolvuli’. Nucleotide sequence data were deposited in the GenBank database under the accession numbers MW037215 for the 16S rRNA gene and MW048762 for the *tuf* gene.

### 4.4. Molecular Typing of ‘Ca. P. solani’ Strains

The *stamp* gene encoding the antigenic membrane protein of ‘*Ca.* P. solani’ was amplified in nested PCR using the StampF/R0 and StampF1/R1 primer pairs following reaction conditions according to Fabre et al. 2011 [43]. Obtained amplicons of all ‘*Ca.* P. solani’-infected bindweed samples were sequenced (MW048763-MW048766) and analyzed using software packages as described above. They were compared with reference *stamp* sequences [39,43] using a BLASTn algorithm to determine the genotype identity.

### 4.5. Design of ‘Ca. P. convolvuli’-Specific Nested PCR

The 900-bp *tuf* gene sequences of *‘Ca*. P convolvuli’ identified in symptomatic *C. arvensis* from Serbia were aligned and compared with reference strains of ‘*Ca.* P. solani’ main *tuf* genotypes tuf-b, tuf-ab (b2) and tuf-a [6,19,53] were retrieved online from the National Center for Biotechnology Information (www.ncbi.nlm.nih.gov) (Figure 2). Significant genetic divergence between these two phytoplasma relatives, i.e., 11.3% pairwise differences, enabled positioning species-specific primers along the full range of the sequence. Hence, the design of primers was targeted at the ends of the gene region to obtain product as long as possible to be comparable in length with other GenBank-available sequences (Table 3). Each primer was designed to be specific to the ‘*Ca.* P. convolvuli’ with most of the nucleotide substitutions positioned at the primers’ 3’-end. Primers characteristics (melting temperature, stability, self-complementarity, etc.) were evaluated using Primer 3 software v. 0.4.0 (https://bioinfo.ut.ee/primer3-0.4.0/) [63,64].

The *tuf* gene specific amplification of ‘*Ca.* P. convolvuli’ was subjected to direct PCR using the Tuf-Con-F1/R1 primer pair (791-bp long fragment) followed by nested PCR with Tuf-Con-F2/R2 primers (725 bp). Both reactions were performed under the same conditions in a 20-µL reaction volume containing High Yield Reaction Buffer A with 1.5 mM MgCl2 (1×), 0.2 mM of each dNTP, 0.4 μM of each primer and 1 U of FastGene *Taq* DNA polymerase (NIPPON Genetics Europe, Dueren, Germany) and 2 µL of DNA extract (50 ng), or of the 20-fold diluted direct PCR product. PCR cycles were performed in a Mastercycler ep gradient S (Eppendorf, Hamburg, Germany) applying the following thermal steps: initial denaturation for 2 min at 95 °C followed by 30 cycles of denaturation step at 95 °C for 40 s, annealing at 55 °C (65 °C in nested reaction) for 40 s and elongation step at 72 °C for 60 s; final elongation was performed at 72 °C for 5 min.

The specificity of the primers and amplification protocol was tested on diverse ‘*Ca.* P. solani’ and ‘*Ca.* P. asteris’ isolates [6,23,39,40,42,44] and was applied to all *C. arvensis* samples from this study. This also included all non-symptomatic bindweed isolates negative for any phytoplasmas as determined by 16S rRNA analysis, which were used as negative controls. The obtained *tuf* gene Tuf-Con-F2/R2 primers delineated nested PCR amplicons of ten selected samples positive for ‘*Ca.* P. convolvuli’ occurrence were verified by sequencing and BLAST comparison to confirm their identity, i.e., the specificity of primers and the identity of sequences.

### 4.6. Design of Duplex SYBR Green-Based Real-Time PCR for Simultaneous Detection of ‘Ca. P. solani’ and ‘Ca. P. convolvuli’

Position of primer sequences for selective co-amplification of ‘*Ca.* P. solani’ and ‘*Ca.* P. convolvuli’ using the SYBR Green-based real-time PCR approach was selected on the basis of *tuf* gene sequence alignment (Figure 2) and analyzed using the melting curve prediction software uMelt Quartz (https://dna-utah.org/umelt/quartz/) [65] to determine the melting point temperatures (Tm) of different segments of aligned sequences. Two qualitatively diverse *tuf* gene regions were determined in relation to GC content and Tm values: the first region closer to the 5′ end of the gene with GC content acc. of 30%, and second region some 100-bp downstream with acc. 40% (Figure 2). Due to 10% difference in GC content between the two segments, predicted melting temperature differences were between 2 °C and 3 °C (depending on chemicals applied in the amplification reaction, i.e., uMelt Quartz parameter settings). Therefore, a set of primers Tuf-Con-Fq/Rq was designed to be specific for selective amplification of ‘*Ca.* P. convolvuli’ on the first segment with a predicted Tm value of 78.0 °C, and a set of primers specific for amplification of ‘*Ca.* P. solani’ on the second segment (Tuf-Sol-Fq/Rq, predicted Tm = 81.0 °C) (Figure 2, Table 3). Primer characteristics were evaluated using Primer 3 software.

Primer specificity and efficiency were first tested and evaluated in a separate single-primer set (singleplex) reactions and later used as a mix of primers in duplex SYBR Green-based real-time PCR. They were tested against bindweed samples in which the presence of ‘*Ca.* P. solani’ or ‘*Ca.* P. convolvuli’ was previously identified and genotyped using *stamp* and *tuf* nested-PCR protocols, respectively. The efficiency and the reproducibility of reaction were tested in 10-fold serial dilutions (5 log_10_ concentrations, from 25 ng to 2.5 pg of plant DNA per reaction), and each dilution was tested in triplicate. The efficiency values were calculated for each reaction using the LinRegPCR method, which minimizes the propagation of errors in quantification due to errors in efficiency calculation caused by either over or underestimation of the baseline values [46]. Moreover, standard curves were constructed and analyzed for each primer set. The slope was calculated for the linear regression line (k) between the log of DNA concentration (ng/reaction) and the estimated threshold cycle (Ct). This slope was used to determine the amplification efficiency, E = (10^−1/k^ − 1), where a value of 1.0 indicates 100% amplification efficiency [66]. The squared regression coefficient was also determined (R^2^). The amplification mixture and conditions were the same in both singleplex and duplex reactions, except that primer concentration were 500 nM each in a single-primer set reactions, while in duplex reactions ‘*Ca.* P. convolvuli’-specific primers were present at 4-fold higher concentration than those specific for ‘*Ca.* P. solani’. This was done to retain sufficient amount of PCR-mixture components for amplification of ‘*Ca.* P. convolvuli’ when occurring in mixed infection with ‘*Ca.* P. solani’ because the Ct values of ‘*Ca.* P. solani’ obtained in singleplex reactions were frequently higher (5–15 Ct) than those obtained for ‘*Ca.* P. convolvuli’ in singleplex reactions.

Duplex SYBR Green-based real-timePCR was performed in a final volume of 10 μL comprising 5 μL of SsoAdvanced™ Universal SYBR^®^ Green Supermix (Bio-Rad Laboratories, Inc., Hercules, CA, USA), ‘*Ca.* P. solani’-specific primers at 100 nM each, ‘*Ca.* P. convolvuli’- specific primers at 400 nM each and 2 μL of DNA extract (50 ng). The reactions were performed in Magnetic Induction Cycler (MIC, Bio Molecular Systems, Upper Coomera, QLD, Australia) under the following amplification conditions: initial denaturation step for 3 min at 98 °C, followed by 35 cycles of 15 s denaturation at 95 °C, 30 s of annealing at 60 °C, and 1 s of elongation at 72 °C. The fluorescence signals were collected during elongation of each cycle. The final step included melting curve analyses (0.3 °C step increments), which were analyzed from 65 °C to 95 °C. The micPCR^©^ software Version 2.6.4 (Bio Molecular Systems, Upper Coomera, QLD, Australia) was used for fluorescence acquisition, estimation of threshold cycles (Ct), efficiency value calculation (LinRegPCR) and standard curve analysis (slope, E and R^2^). Each reaction included at least one blank assay without template, two negative controls corresponding to non-symptomatic plants and two positive controls corresponding to a ‘*Ca.* P. solani’ and ‘*Ca.* P. convolvuli’ positive bindweed samples. All samples were run as triplicates.

To validate the DNA preparation and DNA integrity separate SYBR Green-based real-time PCR amplifications targeting eukaryotic 28S rDNA were performed on the plant DNA gene of all *C. arvensis* samples as an endogenous control. Primers UNI28S-fwd/rev [48] were used under the same amplification conditions described above, except that primer concentrations were 250 nM each, as recommended [48]. The potential for multiplex amplification of all three targets in a single reaction using ‘*Ca.* P. solani’-specific primers (100 nM), ‘*Ca.* P. convolvuli’-specific primers (400 nM) and 28S primers as internal control (100 nM) was also tested both in silico (using uMelt Quartz) and experimentally.

## Figures and Tables

**Table 1:** Sampling localities of symptomatic *Convolvulus arvensis*. GPS coordinates, dates of collection, occurrence of symptoms and number of collected samples.

Region	Location	GPS Coordinates	Date	Symptoms Occurrence	Number of Samples
Eastern Serbia	Donji Milanovac	44°31.501′ N 22°02.500′ E	August 2018	symptomatic	6
			July 2019	symptomatic	10
			August 2019	symptomatic	6
			June 2020	symptomatic	6
				non-symptomatic	6
			August 2020	non-symptomatic	24
	Štubik	44°17.237′ N 22°21.713′ E	July 2019	symptomatic	6
			August 2019	symptomatic	8
				non-symptomatic	12
	Kladovo	44°36.850′ N 22°36.233′ E	September 2020	symptomatic	12
				non-symptomatic	12
Central Serbia	Deč	44°49.292′ N 20°09.354′ E	August 2020	symptomatic	8
				non-symptomatic	12
			September 2020	symptomatic	6
Total					134

**Table 2:** Occurrence and variations in symptoms in diseased *Convolvulus arvensis* and (co)occurrence of ‘*Ca.* P. solani’ and ‘*Ca.* P. convolvuli’ in symptomatic and control plants.

Location	Date	Symptoms ^a^	Number of Samples	Number (%) of Phytoplasma Positive Samples ^b,c^			*Ca*Psol *stamp* Genotype
				*Ca*Psol	*Ca*Pcon	MIX	
Donji Milanovac	August 2018	ba, el, dy	6	0	6	0	/
	July 2019	ba, ul, mrl	10	4	0	6	Rqg50 (10)
	August 2019	ba	6	0	6	0	/
	June 2020	ba, y, lvr, sp	6	0	6	0	/
		control plants	6	0	0	0	/
	August 2020	control plants	24	0	0	0	/
Štubik	July 2019	ba, lvr, sp	6	0	3	3	Rqg50 (3)
	August 2019	ba, y	8	0	3	5	Rqg50 (5)
		control plants	12	0	0	0	/
Kladovo	September 2020	ba, y, ul, is, ssp	12	12	0	0	Rqg50 (8) GGY (2) Rpm35 (2)
		control plants	12	0	0	0	/
Deč	August 2020	ba, ul, y	8	1	2	5	Rqg31 (6)
		control plants	12	0	0	0	/
	September 2020	ba, ul, y	6	0	2	4	Rqg31 (4)
Total			134	17 (25%)	28 (41%)	23 (34%)	Rqg50 (26) Rqg31 (10) GGY (2) Rpm35 (2)
symptomatic/control plants			68/66	17/0	28/0	23/0	

^a^ Symptoms: ba = bushy appearance, control plants = non-symptomatic plants, dy = discrete yellowing, el = elongated leaves, is = internodes shortening, lvr = leaf veins reddening, mrl = marginal reddening of leaves, sp = shoot proliferation, ssp = secondary shoot proliferation, ul = undersized leaves, y = yellowing; ^b^ Phytoplasma identification was performed using the novel duplex SYBR Green-based real-time PCR on *tuf* gene for simultaneous detection of ‘*Ca.* P. solani’ and ‘*Ca.* P. convolvuli’ and confirmed using the ‘*Ca.* P. solani’-specific *stamp* nested PCR protocol [43] and ‘*Ca.* P. convolvuli’-specific *tuf* nested PCR protocol designed in this study; ^c^ *Ca*Psol = ‘*Ca.* P. solani’, *Ca*Pcon = ‘*Ca.* P. convolvuli’; MIX = mixed infection of ‘*Ca.* P. solani’ and ‘*Ca.* P. convolvuli’.

**Table 3:** List of primers, applications and amplicon characteristics for specific and selective identification of ‘*Ca.* P. convolvuli’ and ‘*Ca.* P. solani’ using conventional nested PCR and SYBR Green-based real-time PCR.

Application	Primer Name	Primer Sequence (5′→3′)	Amplicon		
			Length (bp)	GC (%)	Tm (°C) ^a^
‘*Ca.* P. convolvuli’-specific direct PCR	Tuf-Con-F1	TGTAGAATATGAAACTGCAC	791	n.a.	n.a.
	Tuf-Con-R1	ATTGAGGGCGATATTGAGAG			
‘*Ca.* P. convolvuli’-specific nested PCR	Tuf-Con-F2	CCCAGGACATGCTGATTATG	725	n.a.	n.a.
	Tuf-Con-R2	AAAAGGCAGTATGACGTCCC			
‘*Ca.* P. convolvuli’-specific real-time PCR	Tuf-Con-Fq	GGCTCGCCAAGTAGGAGTT	141	31	78.0/78.26 ± 0.11
	Tuf-Con-Rq	GGAATTTCATCGCCTGGGT			
‘*Ca.* P. solani’-specific real-time PCR	Tuf-Sol-Fq	GATCCAGTGCGTGAAGTTGAT	106	41	81.0/80.38 ± 0.12
	Tuf-Sol-Rq	CTTGTCCTCTTTCAACTCTACCA			

n.a. not applicable; ^a^ Melting point temperature (Tm) as predicted using uMelt Quartz/as obtained according to real-time PCR melting curve analysis using the micPCR^©^ software Version 2.6.4 (Bio Molecular Systems) and SsoAdvanced™ Universal SYBR^®^ Green Supermix (Bio-Rad Laboratories, Inc.).

**Table 4:** Performance of the Tuf-Con-Fq/Rq and Tuf-Sol-Fq/Rq primer sets tested in singleplex reactions for amplification of ‘*Ca.* P. convolvuli’ and ‘*Ca.* P. solani’, respectively, evaluated using LinRegPCR method [46].

DNA Concentration (ng/reaction)	‘*Ca.* P. convolvuli’			‘*Ca.* P. solani’		
	Ct Mean ± SD ^a^	Efficiency (E) ^b^	Value of fit (R^2^) ^c^	Ct Mean ± SD ^a^	Efficiency (E) ^b^	Value of Fit (R^2^) ^c^
25	13.70 ± 0.06	0.95–0.98	>0.999	14.14 ± 0.03	0.96–0.98	>0.999
2.5	17.00 ± 0.01	0.91–0.98	>0.999	17.43 ± 0.03	095–0.96	>0.999
0.25	20.56 ± 0.06	0.90–0.95	>0.999	20.92 ± 0.06	0.90–0.97	>0.999
0.025	24.08 ± 0.04	091–0.97	>0.999	24.36 ± 0.04	0.88–0.92	>0.999
0.0025	27.48 ± 0.21	0.91–0.95	>0.999	28.67 ± 0.64	0.87–0.89	>0.999

^a^ Ct, (threshold cycle); ^b^ The amplification efficiency was calculated for each replication using the LinRegPCR algorithm [47]; ^c^ The R-squared value as a quality measure of the linear regression used to calculate amplification efficiency.

## Data Availability

DNA sequences are available in the GenBank database, accession numbers are listed in the Materials and Methods. All other relevant data are within the paper.
